# Curcumin-Etoposide Synergy: Unveiling the Molecular Mechanisms of Enhanced Apoptosis and Chemoresistance Attenuation in Breast Cancer

**DOI:** 10.5812/ijpr-150978

**Published:** 2024-11-05

**Authors:** Bahar Jaberian Asl, Reza Afarin, Mahdi Hatami, Amineh Dehghani Madiseh, Mohammadreza Roshanazadeh, Mojtaba Rashidi

**Affiliations:** 1 Cellular and Molecular Research Center, Medical Basic Sciences Research Institute, Ahvaz Jundishapur University of Medical Sciences, Ahvaz, Iran

**Keywords:** Breast Cancer, Combination, Curcumin, Etoposide, Apoptosis

## Abstract

**Background:**

Combining natural compounds with chemotherapeutic agents has emerged as a promising approach for cancer treatment. Curcumin (Cur), a natural polyphenol, is known for its anti-cancer properties, including the ability to induce apoptosis and arrest cell cycle progression.

**Objectives:**

This study aimed to evaluate the effects of Cur and etoposide (ETO), both individually and in combination, on the induction of apoptosis in breast cancer (BC) cell lines.

**Methods:**

The impact of Cur and ETO on cell proliferation was assessed using MTT viability assays. Apoptosis induction by these drugs was evaluated through Annexin V flow cytometry and caspase-3 and caspase-9 activity assays. Quantitative real-time PCR was employed to measure Bax and Bcl-2 gene expression levels. Western blotting was conducted to determine protein levels of p53, p21, Bax, and Bcl-2.

**Results:**

A non-significant dose of ETO was selected based on MTT assay results and combined with 75 µM of Cur. Curcumin enhanced ETO’s pro-apoptotic effect by increasing caspase activities. The combination of Cur and ETO significantly reduced Bcl-2 gene expression while upregulating Bax expression. Furthermore, treatment with this combination elevated the protein levels of p53, p21, and Bax, compared to ETO or Cur alone, while significantly decreasing Bcl-2 protein levels.

**Conclusions:**

Cur has the potential to amplify ETO-induced apoptosis in BC cells. This combination may offer a promising therapeutic approach for BC.

## 1. Background

In recent years, breast cancer (BC) has become the most common and deadly cancer among women worldwide (1, 2). Breast cancer is a heterogeneous disease, encompassing multiple subtypes (3). Its pathogenesis is influenced by numerous factors, including molecular abnormalities, cellular signaling pathways, and genetic and epigenetic modifications (4). Thus, deciphering the molecular mechanisms and genetic alterations underlying this disease is of paramount importance.

The p53 gene is crucial in cancer prevention, as it arrests the cell cycle and initiates apoptosis in response to DNA damage, thereby preventing carcinogenesis. Dysfunction of p53 is frequently associated with cancer development, with mutations occurring in 50 - 60% of cancers, especially in BC (5, 6). Etoposide (ETO), a widely used chemotherapeutic drug, treats various cancers, including lung, gastric, prostate, and BCs. Etoposide is classified as a topoisomerase inhibitor, primarily acting during the S and G2 phases of the cell cycle. It induces cell death by inhibiting the second phase of topoisomerase II activity, thereby preventing DNA religation (7, 8). However, ETO’s adverse effects, such as neutropenia, myelosuppression, and alopecia, limit its clinical use (9, 10).

Chemoresistance presents a major challenge in chemotherapy, as cancer cells can reduce the efficacy of chemotherapeutic drugs, often rendering them ineffective. It is estimated that a significant proportion of cancer-related deaths result from the failure of chemotherapy (11, 12). Consequently, developing more effective therapeutic strategies for cancer treatment is critically important.

Polyphenols, found in various plants, offer health benefits, including anti-inflammatory and antioxidant effects. Curcumin (Cur), a polyphenol in turmeric, has therapeutic potential against conditions such as cancer, asthma, and Alzheimer’s disease due to its antioxidant and anti-inflammatory properties. It influences several cellular pathways, including PI3K and NF-κB, thereby inhibiting inflammation and gene expression linked to disease progression (13, 14). Although Cur’s anti-cancer properties are well-researched, its potential synergistic effects with chemotherapy drugs like ETO, especially in BC, remain unclear. The mechanisms through which Cur acts, particularly in overcoming p53-mediated chemoresistance, are also not fully understood. Given the challenges of chemoresistance and the severe side effects associated with conventional chemotherapy, there is a pressing need for strategies that enhance treatment efficacy while minimizing toxicity. This study aims to explore the molecular interactions between Cur and ETO, focusing on their combined impact on apoptosis and chemoresistance in BC cells, with the goal of developing safer and more effective cancer therapies.

## 2. Objectives

Many aspects of Cur’s role in cancer treatment remain unexplored. This study aims to investigate whether Cur sensitizes MCF-7 and MDA-MB-231 human BC cell lines to ETO and whether it exhibits synergy with ETO in modulating the expression of the p53 protein, a key cell cycle regulator.

## 3. Methods

### 3.1. Reagents and Cell Culture

MCF-7 and MDA-MB-231 cell lines were obtained from the Pasteur Institute (Tehran, Iran). DMEM medium, fetal bovine serum (FBS), and the antibiotics penicillin and streptomycin were purchased from Idea-Zist (Tehran, Iran), which also provided culture-grade DMSO. Etoposide and Cur were sourced from Sigma-Aldrich, and MTT was procured from Idea-Zist. Cells were maintained in humidified incubators at 37°C with 5% CO_2_. The regular medium was replaced with FBS-free DMEM one day prior to the assay.

### 3.2. MTT Assay

Cells were seeded in separate 96-well plates at a density of 10^4^ cells per well. Fresh DMEM containing either ETO, Cur, or a combination of both replaced the medium. After 24, 48, and 72 hours of incubation, a new medium containing 10% MTT was added to each well. Following a 4-hour incubation, the MTT-containing medium was replaced with DMSO to dissolve the resulting formazan crystals.

### 3.3. Cell Migration Assay

The wound-healing assay was used to assess the effects of Cur and ETO on cell migration at 24 and 48 hours. Cells were cultured to over 90% confluence, and a wound was created using a 5 mL pipette tip. After washing to remove any unattached cells, the remaining cells were treated with 75 µM Cur and 10 µM ETO. Images were taken immediately after treatment, then at 24 hours, and again at 48 hours. Cell migration was analyzed using NIH ImageJ software, and the migration rate was calculated as follows: 


$$Migration\;rate=[(\frac{T0-Th}{T0})]\times 100$$

### 3.4. Quantitative Real-time PCR

Total RNA was extracted using the Yekta Tajhiz Azma kit (FABRK 001, Iran), and purity and integrity were confirmed using a Nanodrop spectrophotometer and gel electrophoresis. cDNA synthesis was performed in a 20 µL reaction volume, followed by 40 cycles of PCR. Real-time PCR measured the relative expression of Bax and Bcl-2 genes using the Amplicon SYBR Green kit, with GAPDH as an internal reference for normalization. Thermal cycling was carried out using the QuantStudio3 PCR instrument (Applied Biosystems, Massachusetts, USA). Primer sequences are listed in Appendix 1 in Supplementary File. A melting curve plot verified the specificity of PCR products, and gene expression levels were quantified based on the average of triplicate experiments.

### 3.5. Western Blot Analysis

Following treatment, cells were lysed in a buffer containing HEPES, NaCl, EDTA, and Triton X-100. Protein concentrations were determined via the BCA assay. Proteins were separated by SDS-PAGE and transferred onto PVDF membranes. After blocking with skim milk in TBST, the membranes were incubated with HRP-conjugated secondary antibodies for 1 hour at room temperature. Protein bands were visualized using the ECL method (Sigma-Aldrich, USA) and quantified by densitometry using ImageJ software.

### 3.6. Flow Cytometry

The apoptotic status of cells was assessed by flow cytometry using the Annexin V-FITC/Propidium Iodide (PI) kit from IQ Products. Following Cur treatment, cells were harvested, trypsinized, and washed with calcium buffer. Cells were then centrifuged, resuspended in calcium buffer containing Annexin V-FITC, and incubated at 4°C for 20 minutes. After this incubation, the buffer was replaced with calcium buffer containing PI, and cells were further incubated for 10 minutes. Apoptosis levels were analyzed using a Becton Dickinson flow cytometer, with a focus on Annexin V-FITC/PI staining patterns.

### 3.7. Assessment of Caspase-3 and Caspase-9 Activity

The activities of caspase-3 and caspase-9 in cell lysates were determined using the Caspase-3 Assay Kit, Colorimetric (Abcam; ab39401, USA). Cells were harvested, resuspended in lysis buffer, and centrifuged to obtain cell extracts. Protein content in each sample was measured, and equal amounts of protein were used for the assays. Caspase-3 and caspase-9 activities were determined by incubating the samples with enzyme-specific colorimetric substrates for 1 hour, followed by absorbance measurement at 405 nm to assess substrate cleavage.

### 3.8. Statistical Analysis

All experiments were performed in triplicate, and data are presented as mean ± standard deviation (SD). Statistical analyses were conducted using GraphPad Prism version 9.0. Group differences were analyzed with one-way analysis of variance (ANOVA), followed by Tukey's post-hoc multiple comparison test. A P-value of less than 0.05 was considered statistically significant.

## 4. Results

### 4.1. Curcumin and Etoposide in Combination Significantly Reduce Breast Cancer Cell Viability

**Figure 1.**
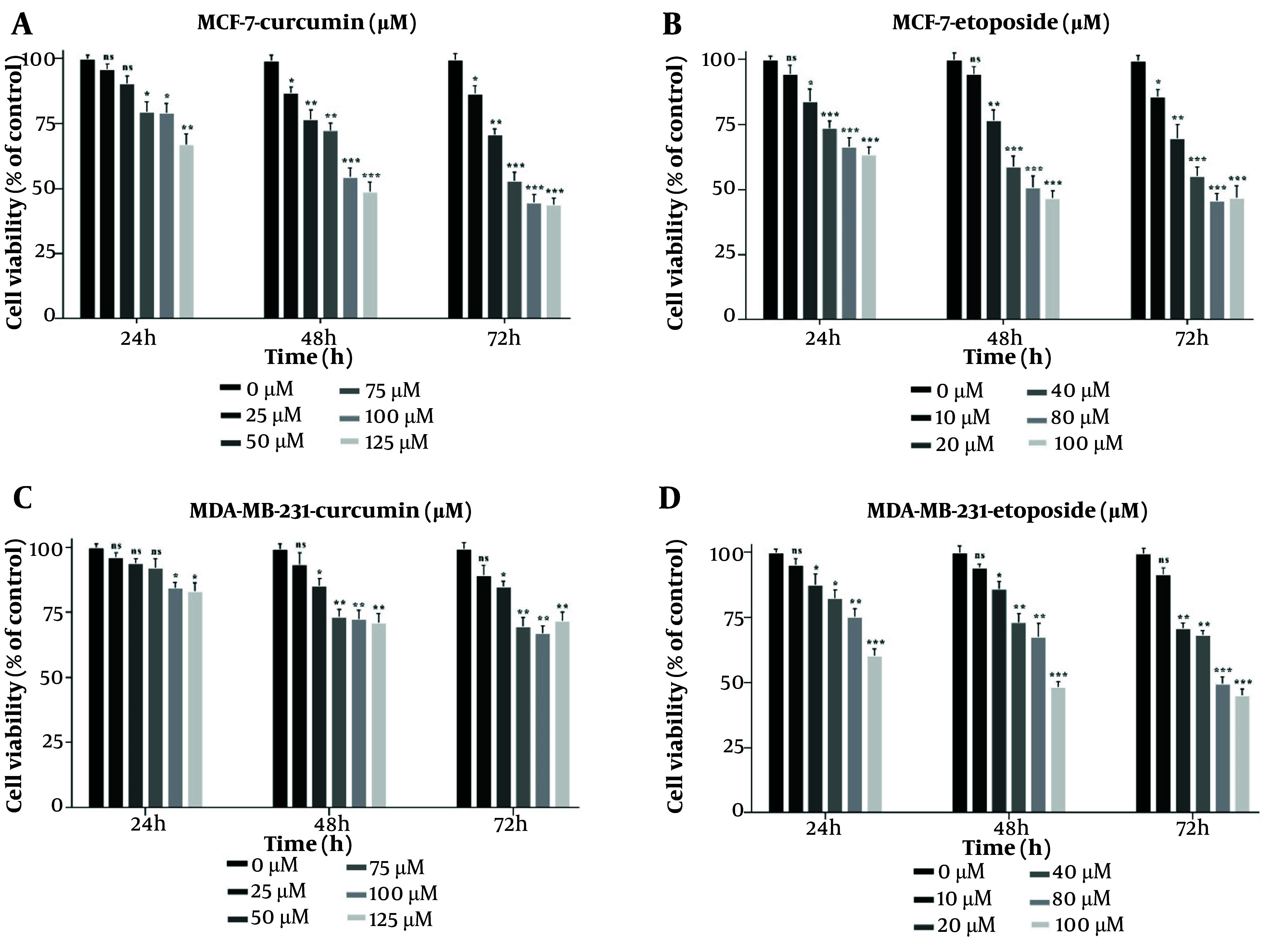
Suppressive effects of curcumin (Cur) and etoposide (ETO) on the viability of MCF-7 and MDA-MB-231 breast cancer (BC) cell lines, assessed using the MTT assay. MCF-7 cells were treated with five different concentrations of Cur (25 - 125 µM) and ETO (10 - 100 µM) for 24, 48, and 72 hours (A and B). MDA-MB-231 cells were treated with five different concentrations of Cur (25 - 125 µM) and ETO (10 - 100 µM) for 24, 48, and 72 hours (C and D). (* P < 0.05, ** P < 0.01, *** P < 0.001 compared to untreated control).

**Figure 2.**
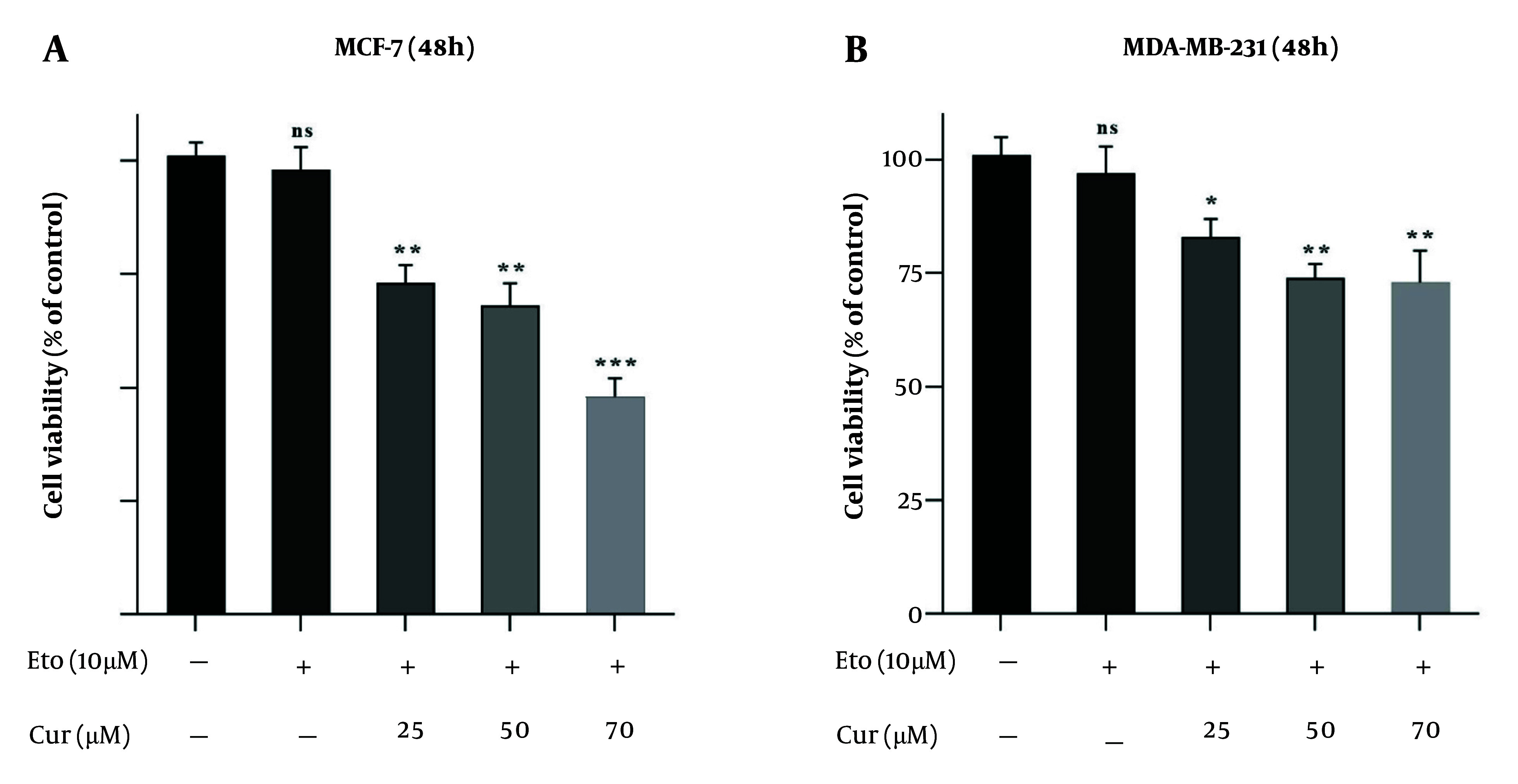
Viability of MCF-7 and MDA-MB-231 cells treated with etoposide (ETO) alone, and with the combination of 10 µM of ETO and 25, 50, or 75 µM of curcumin (Cur). A, MCF-7 cells were treated with combinations of Cur and ETO for 48 hours; B, MDA-MB-231 cells were treated with three different combinations of Cur and ETO for 48 hours. (* P < 0.05, ** P < 0.01, *** P < 0.001 compared to untreated control).

The MTT assay showed that both Cur and ETO individually inhibited the proliferation of MDA-MB-231 and MCF-7 cells in a dose-dependent manner after 48 hours. For Cur, the lowest effective concentrations were 25 µM for MCF-7 and 50 µM for MDA-MB-231, while for ETO, the lowest effective dose was 20 µM for both cell lines. Curcumin had an IC_50_ of 119.2 µM for MCF-7 (P < 0.001, Figure 1A, and B), though no Cur concentration achieved a 50% reduction in viability for MDA-MB-231 cells. ETO's IC_50_ values were 76.4 µM for MCF-7 and 93.6 µM for MDA-MB-231 (P < 0.001, Figure 1C, and D). Results at 72 hours were consistent with those observed at 48 hours. Combining 10 µM ETO with 25, 50, or 75 µM Cur showed significantly enhanced efficacy compared to individual treatments, reducing MCF-7 viability to 73.8%, 68.9%, and 48.6% (P < 0.001, Figure 2A) and MDA-MB-231 viability to 82.6%, 74.7%, and 72.9% (P < 0.01, Figure 2B). The combination of 10 µM ETO with 75 µM Cur yielded the lowest Combination Index (CI) values of 0.43 for MCF-7 and 0.56 for MDA-MB-231, indicating the highest synergy (Appendices 2 and 3 in Supplementary File).

### 4.2. Wound Healing Assay

**Figure 3.**
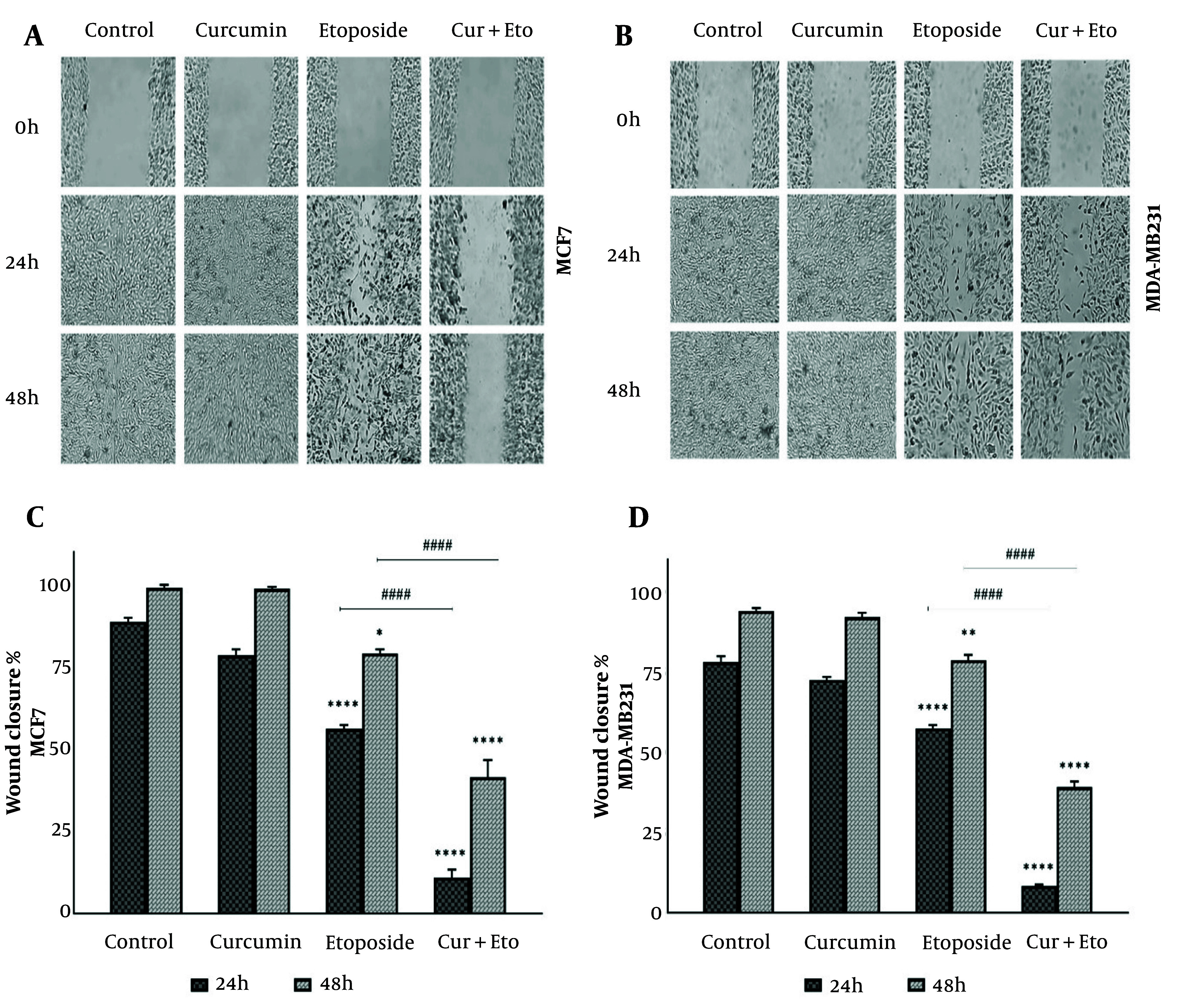
Migration of MCF-7 and MDA-MB-231 cells following treatment with curcumin (Cur) and etoposide (ETO) for 24 and 48 hours. A and B, the process of wound healing in both cell lines, comparing untreated groups with those treated with Cur, ETO, and a combination of both at the 24- and 48-hour marks; C and D, The wound area was quantified using ImageJ, with the resulting values presented as mean ± SD. Statistical significance is indicated as follows: * P < 0.05, ** P < 0.01, **** P < 0.0001, when compared to the untreated control; #### P < 0.0001, when compared to the group treated with ETO.

The effects of Cur, ETO, and their combination on cell migration were evaluated using a wound-healing assay (Figure 3A and B). Breast cancer cells were treated for 24 or 48 hours with Cur, ETO, or a combination of both. The results demonstrated a significant reduction in wound closure speed for both cell lines following ETO treatment alone at 24 and 48 hours (P < 0.0001, Figure 3C and D). The combined treatment further inhibited wound closure at both time points, as indicated by migration indices (P < 0.0001), showing that Cur and ETO together were more effective than ETO alone.

### 4.3. Combined Treatment with Curcumin and Etoposide Significantly Enhances Apoptosis in Breast Cancer Cells

**Figure 4.**
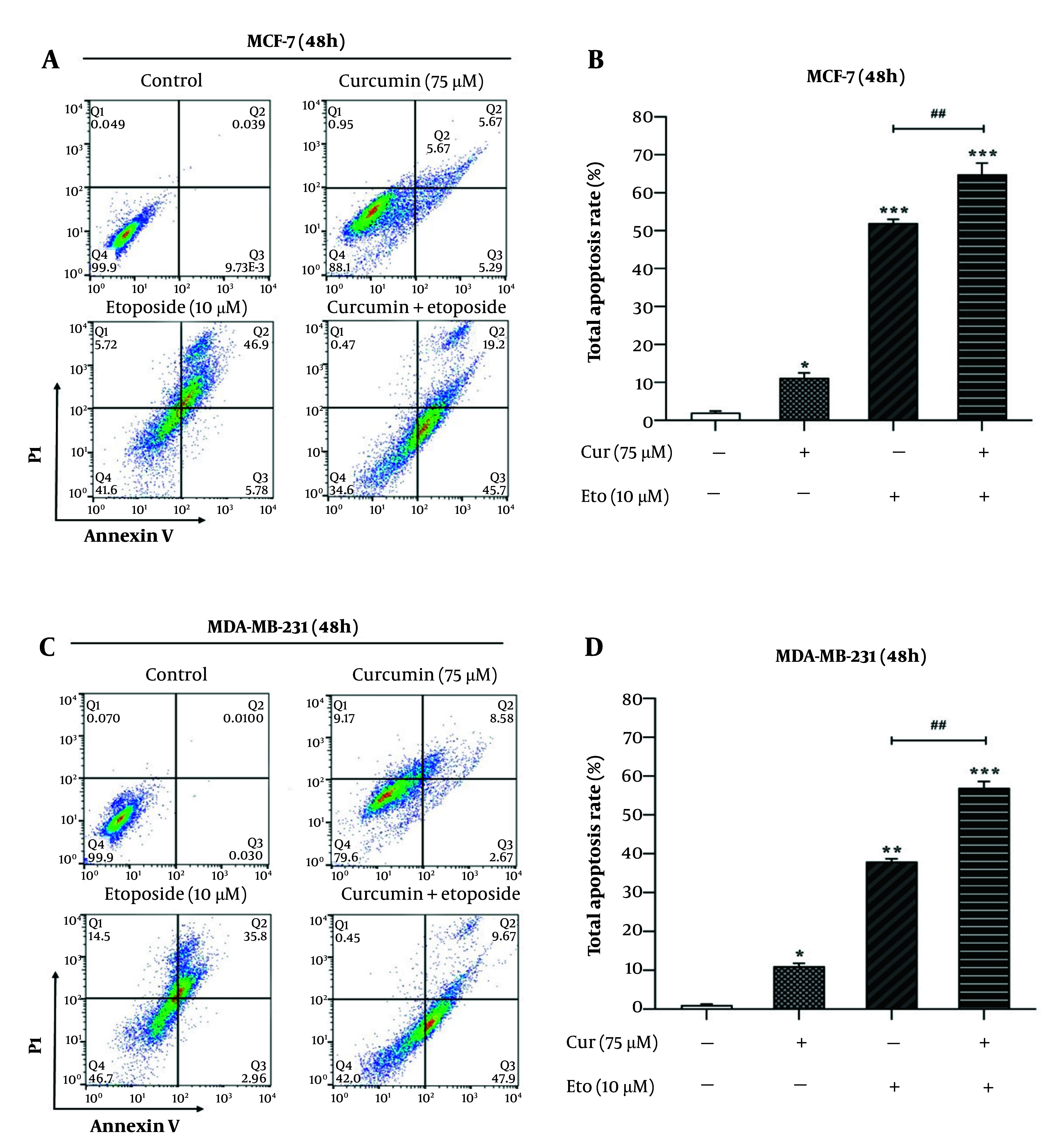
Apoptosis rates of MCF-7 and MDA-MB-231 cells after treatment with 10 µM of etoposide (ETO) combined with 75 µM of curcumin (Cur). A and C, flow cytometry plots for different treatment states for 48 hours; B and D, quantified plots of apoptosis rates of MCF-7 and MDA-MB-231 cells. (*P < 0.05, **P < 0.01, ***P < 0.001 compared to untreated control; ##P < 0.01 compared to the ETO-treated group).

Flow cytometry analysis using Annexin V/PI staining revealed that ETO alone, but not Cur, led to a substantial increase in apoptotic cell death in both MCF-7 and MDA-MB-231 cells. However, the combination of Cur and ETO resulted in a markedly greater apoptotic effect than either agent alone. In the MCF-7 and MDA-MB-231 control groups, baseline apoptosis rates were 2.82% (P < 0.001) and 2.53% (P < 0.001), respectively. When treated individually, Cur and ETO increased these apoptosis rates to 13.1% (non-significant) and 19.62% in MCF-7 cells, and to 6.71% (non-significant) and 15.02% in MDA-MB-231 cells, respectively. In contrast, the combined treatment with ETO and Cur significantly elevated apoptosis levels to 35.2% in MCF-7 cells (Figure 4A and B) and 26.7% in MDA-MB-231 cells (Figure 4C and D).

### 4.4. Synergistic Enhancement of Apoptosis Markers by Curcumin-Etoposide Combination

**Figure 5.**
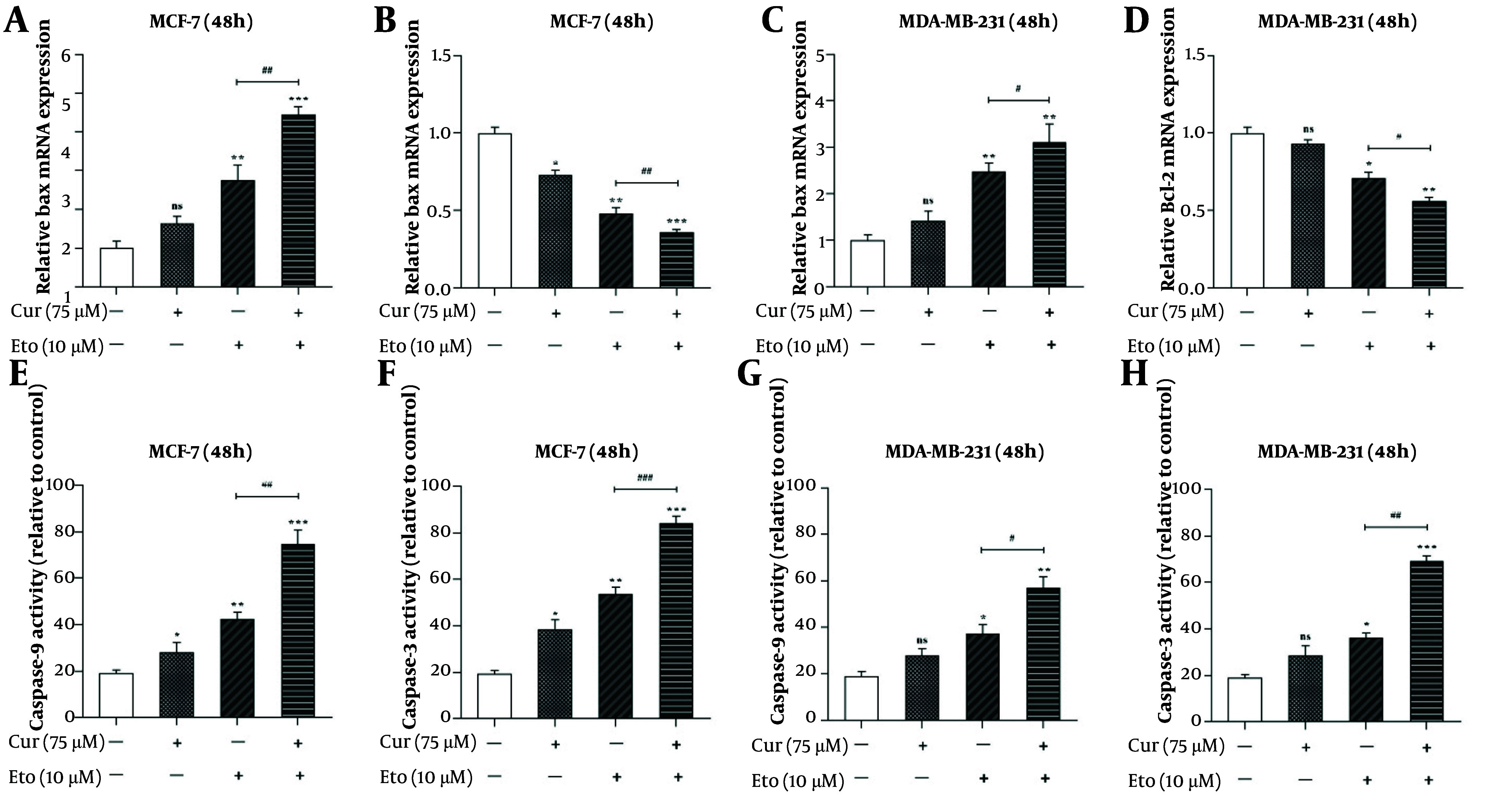
Relative mRNA expressions of Bax, Bcl-2, and activities of caspase-9 and caspase-3 in curcumin (Cur) and etoposide-treated MCF-7 and MDA-MB-231 cells. A, effects of the combination of Cur and etoposide (ETO) on the expression of the Bax gene in MCF-7 cells; B, effects of the combination of Cur and ETO on the expression of the Bcl-2 gene in MCF-7 cells; C, effects of the combination of Cur and ETO on the expression of the Bax gene in MDA-MB-231 cells; D, effects of the combination of Cur and ETO on the expression of the Bcl-2 gene in MDA-MB-231 cells; E and F, effects of Cur and ETO on the activities of caspase-9 and caspase-3 in MCF-7 cells; G and H, effects of Cur and ETO on the activities of caspase-9 and caspase-3 in MDA-MB-231 cells. (* P < 0.05, ** P < 0.01, *** P < 0.001 compared to untreated control; # P < 0.05, ## P < 0.01, ### P < 0.001 compared to the ETO-treated group).

The combination of Cur and ETO significantly increased Bax expression and decreased Bcl-2 expression in both cell lines. In MCF-7 cells, individual treatments resulted in a 1.64-fold increase in Bax expression with Cur (non-significant) and a 2.76-fold increase with ETO, alongside reductions in Bcl-2 by 0.73-fold and 0.48-fold, respectively. In MDA-MB-231 cells, Bax expression increased by 1.42-fold with Cur (non-significant) and 2.48-fold with ETO, while Bcl-2 decreased to 0.93-fold (non-significant) and 0.71-fold, respectively. The Cur-ETO combination produced a more pronounced increase in Bax expression (4.46-fold in MCF-7, P < 0.001; 3.1-fold in MDA-MB-231, P < 0.01) and a greater reduction in Bcl-2 (0.36-fold in MCF-7, P < 0.001; 0.57-fold in MDA-MB-231, P < 0.01) compared to individual treatments (Figure 5). 

Caspase-3 and caspase-9 activities were also enhanced in MCF-7 cells treated with either Cur or ETO alone, with Cur increasing these activities to 38.6% and 28.2% and ETO to 53.7% and 42.4% (Figure 5E and F). In MDA-MB-231 cells, Cur had no significant effect on caspase activity, while ETO increased activities to 36.3% and 37.2% (Figure 5G and H). The combination of Cur and ETO further amplified caspase-3 and caspase-9 activities in MCF-7 cells to 84.3% (P < 0.001) and 74.6% (P < 0.001), and in MDA-MB-231 cells to 69.1% (P < 0.001) and 56.8% (P < 0.01), respectively (Figure 5). 

### 4.5. Curcumin and Etoposide Synergistically Increase p53, p21, and Bax Levels While Decreasing Bcl-2

**Figure 6.**
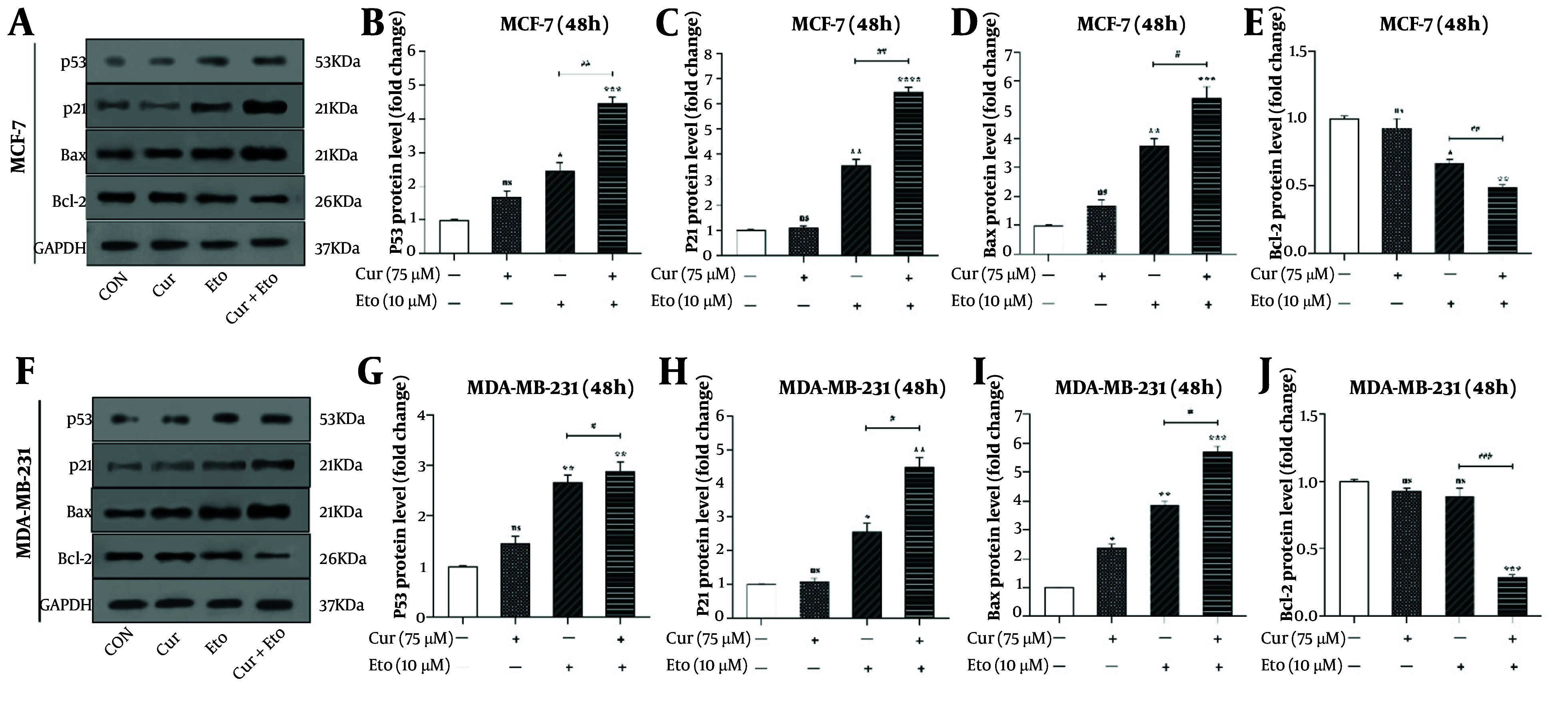
p53, p21, Bax, and Bcl-2 protein levels in curcumin (Cur) and etoposide-treated MCF-7 and MDA-MB-231 cells, assessed through western blotting. A and F, the effects of Cur and etoposide (ETO) on the protein expression levels of p53, p21, Bax, and Bcl-2; B and G, quantified plots of the impacts of Cur and ETO, individually and in combination, on the relative protein expression of p53 in MCF-7 and MDA-MB-231 cells; C and H, quantified plots of the impacts of Cur and ETO, individually and in combination, on the relative protein expression of p21 in MCF-7 and MDA-MB-231 cells; D and I, quantified plots of the impacts of Cur and ETO, individually and in combination, on the relative protein expression of Bax in MCF-7 and MDA-MB-231 cells; E and J, quantified plots of the impacts of Cur and ETO, individually and in combination, on the relative protein expression of Bcl-2 in MCF-7 and MDA-MB-231 cells. (* P < 0.05, ** P < 0.01, *** P < 0.001, **** P < 0.0001 compared to untreated control; # P < 0.05, ## P < 0.01, ### P < 0.001 compared to the ETO-treated group).

The effects of Cur and ETO on the proteins p53, p21, Bax, and Bcl-2 were also assessed (Figure 6). Etoposide significantly elevated the expression of p53, p21, and Bax in both MCF-7 and MDA-MB-231 cells, while Cur had minimal effects individually. Specifically, ETO increased p53, p21, and Bax by 2.46- and 2.67-fold, 3.54- and 2.56-fold, and 3.76- and 3.86-fold, respectively, in MCF-7 and MDA-MB-231 cells. Cur caused modest increases in p53 and Bax, by 1.68- and 1.46-fold for p53 and 1.63- and 2.38-fold for Bax. Additionally, ETO decreased Bcl-2 expression to 0.67-fold in MCF-7, with no significant effect in MDA-MB-231 cells.

The combined treatment of Cur and ETO further enhanced the expression of p53 (4.48-fold in MCF-7, P < 0.001; 2.89-fold in MDA-MB-231, P < 0.01), p21 (6.48-fold in MCF-7, P < 0.001; 4.46-fold in MDA-MB-231, P < 0.01), and Bax (5.42-fold in MCF-7, P < 0.001; 5.72-fold in MDA-MB-231, P < 0.001), while significantly reducing Bcl-2 expression to 0.49-fold in MCF-7 P < 0.01) and 0.29-fold in MDA-MB-231 P < 0.001) (Figure 6). 

## 5. Discussion

Chemotherapy has long been the primary treatment for cancer, although it often results in severe side effects (15, 16). Recent research has begun to explore combining chemotherapeutic agents with natural compounds, such as plant polyphenols, to help reduce these side effects. The rationale is that combining a chemotherapeutic drug with a less toxic natural compound may allow for lower doses of each drug, thus reducing overall toxicity while enhancing treatment effectiveness (17, 18). This approach is particularly valuable, as certain cancers develop resistance to chemotherapy over time (19, 20). Etoposide is a widely used chemotherapeutic agent, but it also encounters issues of resistance in cancers like neuroblastoma, small cell lung cancer, and BC (21, 22). Combining ETO with natural compounds targets processes such as apoptosis, cell migration, and angiogenesis. Apoptosis, in particular, involves intrinsic and extrinsic pathways, with key regulators such as p53, Bcl-2 proteins, and caspases, which are essential targets in cancer therapy (23, 24).

Natural compounds influence critical signaling pathways, including Erk1/2, PI3K/Akt, and NF-κB, thereby impacting cellular mechanisms like oxidative stress and apoptosis (25). Curcumin, a natural polyphenol, exhibits anti-inflammatory, antioxidant, and pro-apoptotic properties (26). In this study, we evaluated the effects of ETO and Cur on apoptosis in MCF-7 and MDA-MB-231 BC cell lines. While high doses of ETO and Cur individually reduced cell viability, their combination significantly enhanced ETO’s efficacy. Specifically, the combination lowered the 48-hour IC_50_ of ETO from 80 µM to 10 µM in MCF-7 cells, although it did not reach 50% inhibition in MDA-MB-231 cells. These findings indicate that Cur potentiates ETO’s effect and may help overcome chemoresistance (27, 28). This effect might be attributed to Cur's modulation of various cellular pathways that contribute to tumor cell survival, including the inhibition of NF-κB and STAT3 signaling pathways, both of which play crucial roles in cell proliferation and survival (29).

The wound-healing assay demonstrated that the combination of Cur and ETO significantly inhibited wound closure at 24 hours in both cell lines, suggesting a reduction in migration. This finding is consistent with Mohammed et al., who reported that Cur impedes the migration of MDA-MB-231 cells (30), and Hamsa et al., who found that ETO similarly inhibits migration (31). The synergistic inhibition of wound closure by Cur + ETO could be attributed to their combined action on pathways including the inhibition of NF-κB activation, which plays a crucial role in regulating genes involved in inflammation, proliferation, and metastasis (32). Etoposide primarily functions as a topoisomerase II inhibitor, inducing DNA damage and apoptosis. However, it also affects cell adhesion molecules and cytoskeletal dynamics, both essential for cell migration (33).

The combination of Cur + ETO increased apoptosis rates by 15.8% in MCF-7 cells and 11.5% in MDA-MB-231 cells compared to ETO alone. Curcumin alone at 75 µM did not significantly impact apoptosis; however, in combination with ETO, it significantly enhanced apoptosis, indicating a synergistic effect. Dhima et al. observed that Cur upregulates pro-apoptotic factors such as p53, Cdk inhibitors, and caspases, as well as inducing cell cycle arrest, potentially complementing the effects of ETO (34). In this study, Cur did not significantly increase p53 levels on its own. Etoposide showed higher effectiveness in MCF-7 cells than in MDA-MB-231 cells, possibly due to the lower malignancy and chemoresistance in MCF-7 cells. The molecular subtypes of these cell lines MCF-7’s luminal type and MDA-MB-231’s triple-negative breast cancer (TNBC) may contribute to these differential responses, though further investigation is needed to understand the link (35).

The observed synergy in promoting apoptosis may be attributed to the combined modulation of Bcl-2 and Bax proteins, along with the significant increase in caspase-3 and caspase-9 activities. Etoposide induces DNA damage, which activates p53-dependent apoptotic pathways (36). Curcumin may further sensitize cells to ETO-induced apoptosis by impacting NF-κB and other survival pathways, as evidenced by the substantial reduction in Bcl-2 levels and increase in Bax levels, which establish a pro-apoptotic environment and enhance caspase activity in both cell lines (37).

Our results indicate that Cur enhances the effects of ETO, leading to a greater decrease in Bcl-2 and an increase in Bax expression, particularly in MCF-7 cells. Additionally, the levels of p53, p21, and Bax proteins increased significantly with ETO and ETO + Cur treatments, while Bcl-2 decreased, with a nearly twofold increase in p53 levels, especially in MCF-7 cells. The effects were less pronounced in MDA-MB-231 cells. These findings align with Oak et al. (38), who demonstrated that Cur treatment induces ubiquitination and destabilization of mutant p53 (Mutp53) but not wild-type p53 (WTp53) in cancer cells. The synergy between Cur and ETO in our study is likely due to the destabilization of Mutp53 by Cur, increasing apoptosis through upregulation of Bax and tumor suppressor proteins (p53 and p21) and downregulation of Bcl-2. This effect varies between MCF-7 and MDA-MB-231 cells, with MCF-7 cells showing greater sensitivity due to higher WTp53 levels and lower Mutp53 aggregation, while the more complex effect observed in MDA-MB-231 cells could be due to possible Mutp53 destabilization. Ultimately, the combination of Cur and ETO may help overcome resistance mechanisms, restoring cell cycle arrest and apoptosis in tumor cells (39, 40).

### 5.1. Conclusions 

Our study underscores the synergistic enhancement of ETO efficacy by Cur in BC cells, particularly in MCF-7 cells. The combination reduces cell viability and inhibits wound closure, suggesting a possible reduction in metastatic potential. This synergy is attributed to Cur's modulation of multiple cellular pathways, including the destabilization of Mutp53, particularly in MDA-MB-231 cells, which promotes apoptosis and mitigates chemoresistance. Increased expression of Bax, p53, and p21, along with reduced Bcl-2 expression and significant increases in caspase-3 and caspase-9 activities, supports the pro-apoptotic environment created by this combination. The differential responses observed between MCF-7 and MDA-MB-231 cells highlight the importance of molecular subtypes in determining treatment efficacy. Overall, the combination of Cur and ETO represents a promising strategy to enhance apoptosis and reduce chemoresistance in BC.

ijpr-23-1-150978-s001.pdf

## Data Availability

Research data from this study are available from the corresponding author upon reasonable request.
