# Supplementary material for: Curcumin-Etoposide Synergy: Unveiling the Molecular Mechanisms of Enhanced Apoptosis and Chemoresistance Attenuation in Breast Cancer
Source: Iran J Pharm Res. 2024 Nov 5;23(1):e150978. doi: 10.5812/ijpr-150978 (PMC11742740; doi:10.5812/ijpr-150978)
Supplement: ijpr-23-1-150978-s001.pdf [file ijpr-23-1-150978-s001.pdf]

Appendix 1.The primer sequences of gene. Displays the primer sequences utilized in the real-time PCR assays. The amount of each gene was normalized to the amount of GAPDH.

| Gene | Forward Primer | Reversed Primer |
|---|---|---|
| Bax | CAGGGGCCCTTTTGCTTCA | ACGGCGGCAATCATCCTCT |
| Bcl-2 | GGATAACGGAGGCTGGGATG | TGACTTCACTTGTGGCCCAG |
| GAPDH | ACCCTTAAGAGGGATGCTGC | CCCAATACGGCCAAATCCGT |

Appendix 2. Combination Index (CI) between Curcumin and Etoposide in MCF-7 cells

| | **Curcumin (25 µm)** | **Curcumin (50 µm)** | **Curcumin (75 µm)** |
|---|---|---|---|
| **Etoposide (10 µm)** | 0.87 | 0.68 | 0.43 |

MCF-7 cells incubated with 10 nM of Etoposide and various concentrations of Curcumin. CI was calculated by CompuSyn software. CI < 1.0 represents synergism, CI = 1.0 represents an additive, and CI >1.0 represents antagonism.

Appendix 3. Combination Index (CI) between Curcumin and Etoposide in MDA-MB-231 cells

| | **Curcumin (25 µm)** | **Curcumin (50 µm)** | **Curcumin (75 µm)** |
|---|---|---|---|
| **Etoposide (10 µm)** | 0.91 | 0.74 | 0.56 |

MDA-MB-231 cells incubated with 10 nM of Etoposide and various concentrations of Curcumin. CI was calculated by CompuSyn software. CI < 1.0 represents synergism, CI = 1.0 represents an additive, and CI >1.0 represents antagonism.
